# Conservative Management of Keratocystic Odontogenic Tumors of Jaws

**DOI:** 10.1100/2012/680397

**Published:** 2012-02-14

**Authors:** Nurhan Güler, Kemal Şençift, Özge Demirkol

**Affiliations:** Department of Oral and Maxillofacial Surgery, Faculty of Dentistry, Yeditepe University, Bagdat Cad. No: 238 Goztepe, Istanbul, Turkey

## Abstract

*Purpose*. The aim of this study was to evaluate different surgical treatment methods for keratocystic odontogenic tumors (KCOTs) and the outcome of those treatments over a 9-year period. *Patients and Methods*. A retrospective review was performed on 43 KCOTs in 39 patients. In radiographic evaluations for diagnosis, follow ups and before and after treatment, panoramic, 3D CT and MR images were used. The three groups of different surgical treatment were (1) enucleation for small unilocular lesions without certainty of histology; (2) enucleation with Carnoy's solution, for small unilocular lesions after previous histological confirmation of KOCT; (3) marsupialization followed by enucleation with Carnoy's solution implemented for large often multilocular KCOTs with intact or destruction of cortical bone without infiltration of neighbouring tissue. *Results*. 43 KCOT cases were mostly localized in mandible (76.7%), radiologically unilocular (72%), and parakeratocysts (88.4%). Inflammation and satellite cysts (daughter cysts) were detected histopathologically in 14 (32.5%) and 7 (16.3%), respectively. Among the 43 cysts, 20 (46.5%) were associated with the impacted third molar and of 21 (48.8%) was in tooth bearing area, and 5 (11, 6%) located on edentulous areas. It was located mostly in the anterior region of maxilla (90%) and in mandibular molar and ramus (62.8%). The treatments of KCOTs were 18 (41.9%) for group 1, and 10 (23.3%) group 2, and 15 (34.8%) group 3. A statistically significant relationship was found between the radiographic appearance and treatment methods (*P* = 0.00). No recurrence was found on 40.54 ± 23.02 months follow up. *Conclusion*. We concluded that successful treatment methods were enucleation and Carnoy's solution in small lesions and marsupialization in lesions that have reached a very large size, but because KCOT was observed in second decade mostly, long-term follows up are suggested.

## 1. Introduction

Keratocystic odontogenic tumor (formerly odontogenic keratocysts) (KOCT) is a unique cyst because of its locally aggressive behavior, high recurrence rate, and characteristic histological appearance and comprises approximately 11% of all cysts of the jaws [1]. The radiographic appearance is one of a unilocular or multilocular well-circumscribed radiolucent lesion with scalloped and corticated margins. Involvement of an impacted tooth has been reported in 25% to 40% of cases [2]. Radiographically, displacement of impacted or erupted teeth, root resorption, root displacement, or extrusion of erupted teeth may be evident [3]. KCOTs may occur in any part of the jaws with a considerable predilection for the posterior body of the mandible and ascending ramus with a peak incidence in patients between 10 and 30 years of age and a slight male predominance [4–6]. A noticeable number of cases, however, are diagnosed incidentally during routine dental examination, and the frequency of such cases has been reported to range from 5.5 to 42.5% [4, 7, 8]. Clinically, the parakeratinizing lesions are characterized by aggressive growth and a tendency to recur after surgical treatment. They show increased mitosis in the cystic epithelium, together with a potential for budding of the basal layer and the presence of daughter cysts in the cystic wall. In addition, they show an association with nevoid basal cell carcinoma syndrome [9, 10]. Consequently, in 2005, WHO Working Group considered the KCOT parakeratinizing variant to be a cystic neoplasm and recommended the more descriptive term “keratocystic odontogenic tumor” (KCOT) [11]. The keratocyst is believed to originate from remnants of the dental lamina, following features such as a thin, bandlike lining of stratified squamous epithelium, a spinous cell layer 8 to 10 cells in thickness and a corrugated keratinized lining, a thin, inflammation-free connective tissue capsule, and a lumen-containing varying amounts of desquamated keratin. A parakeratin lining predominates in the majority of KCOTs ranging from 83% to 97% [12–15] and exhibits potential for local destruction and extension into adjacent tissues, rapid growth, a higher rate of recurrence, and a tendency for multiplicity. This may be because of its active epithelial proliferation, prostaglandin-induced bone resorption via interleukins (ILs) and tumors necrosis factors, and active collagenases in the fibrous cystic wall [16–18].

Although various therapies ranging from conservative methods, such as enucleation (with or without curettage), decompression, and marsupialization to aggressive treatments which include peripheral ostectomy with rotary instruments, cryotherapy with liquid nitrogen, and application of Carnoy's and jaw resection have been reported in the literature, the universally accepted approach remains undecided. The eradication of the cyst and the reduction of risks of recurrence and surgical morbidity are main goals of all techniques [7, 18–21]. The type of treatment rendered is controversial, but depends on several factors including patient age, location and size of the lesion, and whether the KCOT is primary or recurrent [22]. However complete removal of the KCOT can be difficult because of the thin friable epithelial lining. Although the presence of satellite cysts and rests of odontogenic epithelium have often been suggested as reasons for KCOT recurrence, the type of epithelial keratinization appears to play a key factor in the tendency for recurrence [14, 15].

Computerized tomography (CT) is a very valuable tool in diagnosis and treatment planning, providing the ability to eliminate image superimposition and the present real dimension values, and the reconstructing images in various planes including 3 dimensions (3D) [23]. Multidetector (MDCT) or cone-beam CT (CBCT) has excellent spatial and high contrast resolution and allows the production of maximum intensity projections and 3D volume rendered images. MDCT has the advantage over CBCT of demonstrating soft-tissue detail and allowing accurate measurement of attenuation. Soft-tissue visualisation allows detection of dense keratin debris in KCOTs and allows distinction between cysts and solid tumours. The extent of a lesion's relationship to teeth, root resorption, internal structure, cortical expansion and erosion, the boundary of a lesion, and the presence of multiple lesions can all be evaluated [24].

The aim of this study was to evaluate different surgical treatment methods for KCOTs and the outcome of those treatments over a 9-year period. 

## 2. Patients and Methods

We retrospectively analyzed all sporadic KCOTs patients treated in the Department of Oral and Maxillofacial Surgery, Yeditepe University, faculty of dentistry from 2001 to 2010. Approval for the study was obtained from the local ethics committee, and all patients signed informed consent. The data including patient age and gender, site of involvement, clinical manifestation and diagnosis, radiographic features, microscopic findings (orthokeratocyst or parakeratocyst, the presence of satellite cysts and inflammation), association with impacted teeth, treatment modalities, recurrences, the mean follow up, and complications were recorded. To evaluate the cyst on radiographic examinations for diagnosis follow up and recall, panoramic radiograph were used for small KCOTs while MDCT, 3D CT and magnetic resonance imaging (MRI) were used before and after treatment of KCOTs and follows up in multilocular and large lesions for detecting hard- and soft-tissue involvement of cysts. The three groups of different surgical treatment were (1) enucleation for small unilocular lesions without certainty of histology; (2) enucleation with Carnoy's solution also applied for small unilocular lesions after previous histological confirmation of KOCT; (3) marsupialization followed by enucleation with Carnoy's solution implemented for large often multilocular KCOTs with intact or destruction of cortical bone without infiltration of neighboring tissue.

## 3. Results

The patient characteristics are shown in Table 1. The study consisted of 43 KCOTs in 39 patients with a mean 40.59 ± 17.39 years ranging from 15 to 87 years, 23 males (59%) and 16 females (41%). The male to female ratio was 1.4 : 1. While bilateral KCOTs located in one patient's maxilla, another one patient had a three KCOTs, which was of 2 located in maxilla and one in mandible. Radicular cyst (32.6%) and dentigerous cyst (21%) were mostly clinically diagnosed before histopathologic examination (Table 2).

KCOT was mostly observed between 20 to 29 years (32.5%), and a negative correlation was found between the age and KCOT with tooth bearing area (*P* = 0.002) (Table 3).

43 KCOT cases were mostly localized in mandible (76.7%), radiologically unilocular (72%), and parakeratocysts (88.4%). Inflammation and satellite cysts (daughter cysts) were detected histopathologically in 14 (32.5%) and 7 (16.3%) of KCOTs, respectively. All inflammation was associated with parakeratocysts. Among the 43 cysts, 20 (46.5%) were associated with the impacted third molar and of 21 (48.8%) was in tooth bearing area, and 5 (11,6%) located on edentulous areas. It was located mostly in the anterior region of maxilla (90%) and in mandibular molar and ramus (62.8%).

The distribution of the radiologic and microscopic findings of KCOTs according to the age groups is seen in Table 3. The most unilocular (29%) and multilocular (41.6%) appearance and parakerotocyst (34%) were recorded at second decade. There is no correlation between the age groups and radiologic and microscopic findings of KCOTs (*P* > 0.05).

The distribution of localization of KCOTs according to the radiographic features is shown in Table 4. The most unilocular appearance of lesions was seen in anterior-premolar region of maxilla (25.8%) and molar ramus of mandible (54.8%) while multilocular was mostly seen in molar-ramus area of mandible (83%).

The distribution of microscopic findings of KCOTs according to the radiographic features is shown in Table 5. 29 (93.5%) KCOTs with parakeratocyst were seen in unilocular appearance. The radiographic appearances of KCOTs with orthokeratocyst were equally distributed.

**Figure 1:**
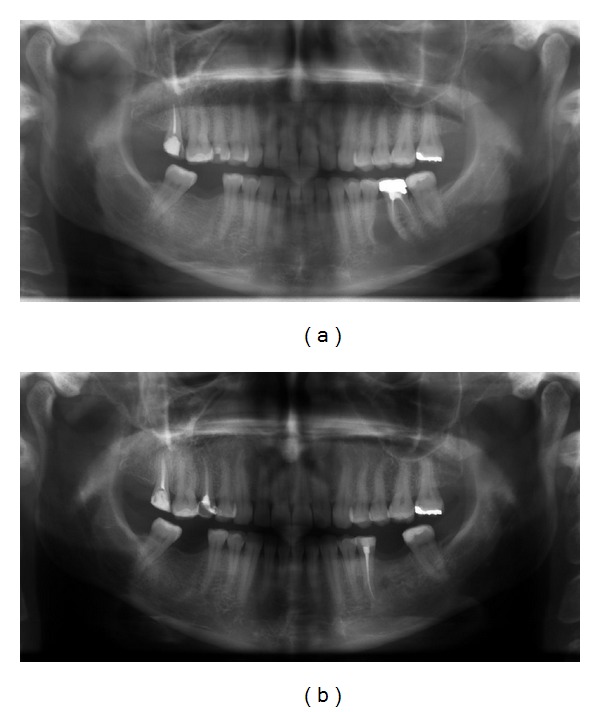
Panoramic view of the KCOT located to between the left lower second premolar and first molar roots (a) and postoperative appearance of lesion treated by enucleation after 2 years (b).

**Figure 2:**
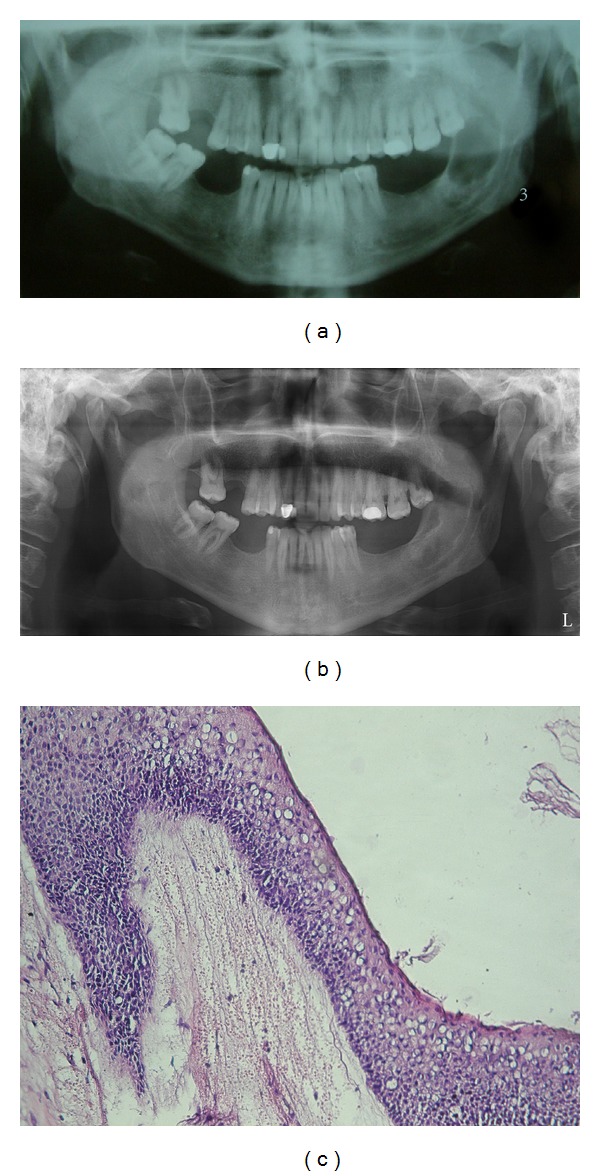
Panoramic view of the KCOT located to the left mandibular ascending ramus (a) and postoperative appearance of lesion treated with enucleation and Carnoy's solution after 5.5 years (b) and the basal layer of epithelium is composed of hyperchromatic cuboidal and columnar cells and parakeratinized epithelial cells border the lumen (Hex400) (c).

**Figure 3:**
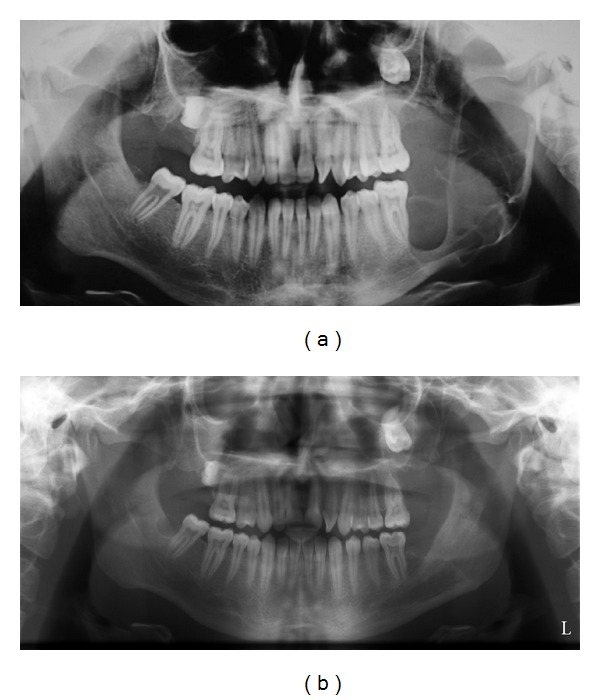
Panoramic view of the KCOT located at the left mandibular ascending ramus with a multilocular appearance (a) and postoperative appearance of the lesion treated by marsupialization followed by enucleation with Carnoy's solution after 3 years (b).

**Figure 4:**
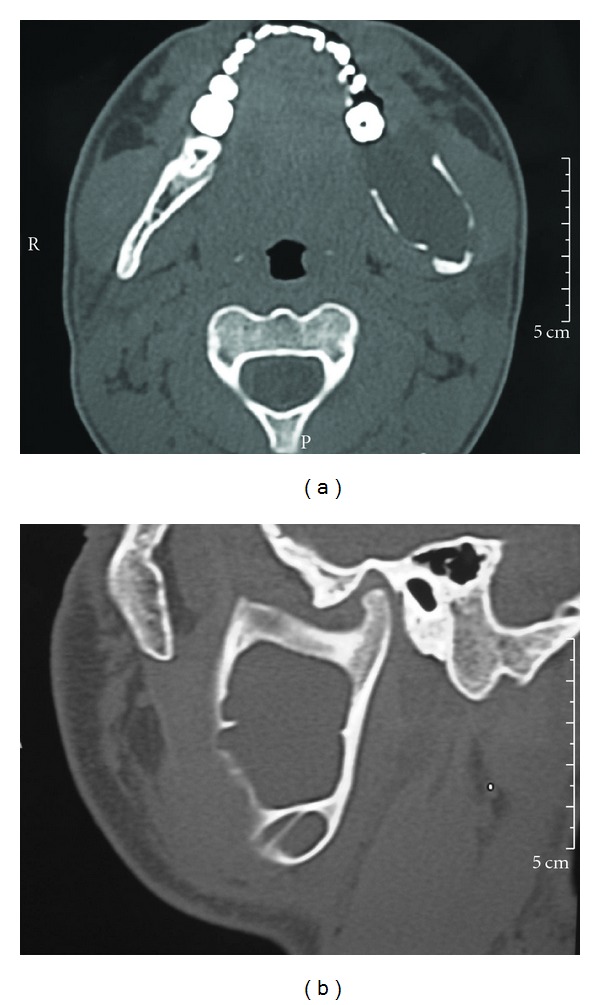
CT axial (a) and sagittal (b) images demonstrate the lingual and buccal cortical expansion and erosion.

**Figure 5:**
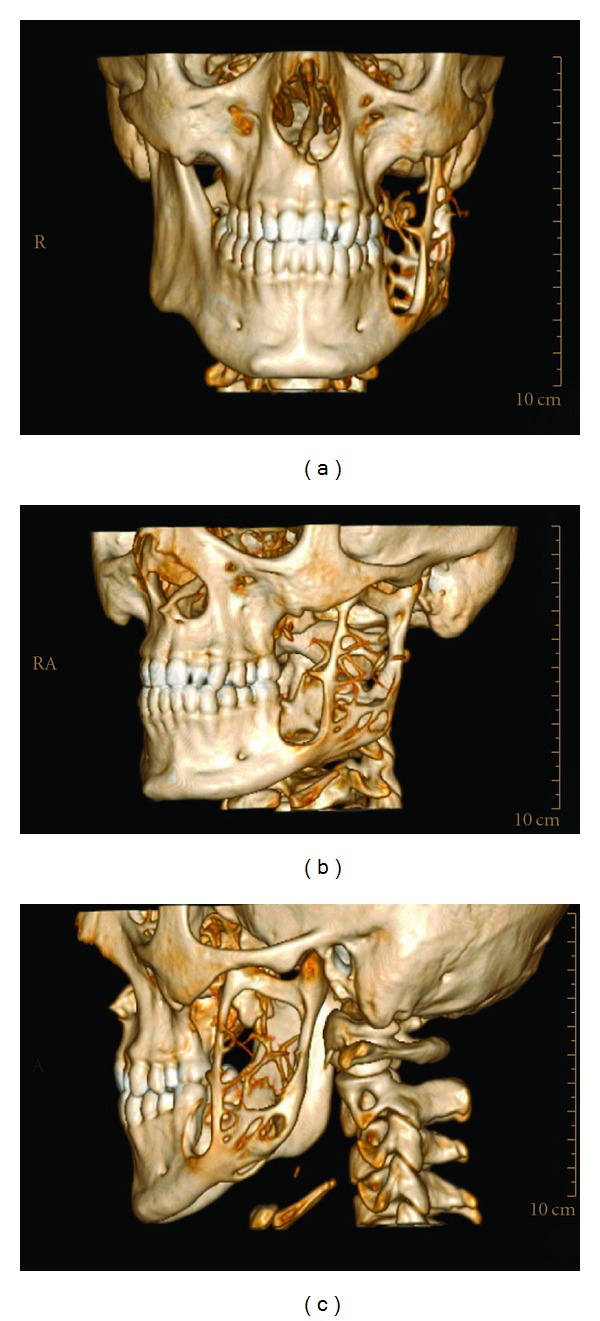
3D CT frontal (a) sagittal (b,c) images demonstrate the lingual, buccal cortical and basis of mandibular erosion with a multilocular bony defect like soup bubble appearance.

**Figure 6:**
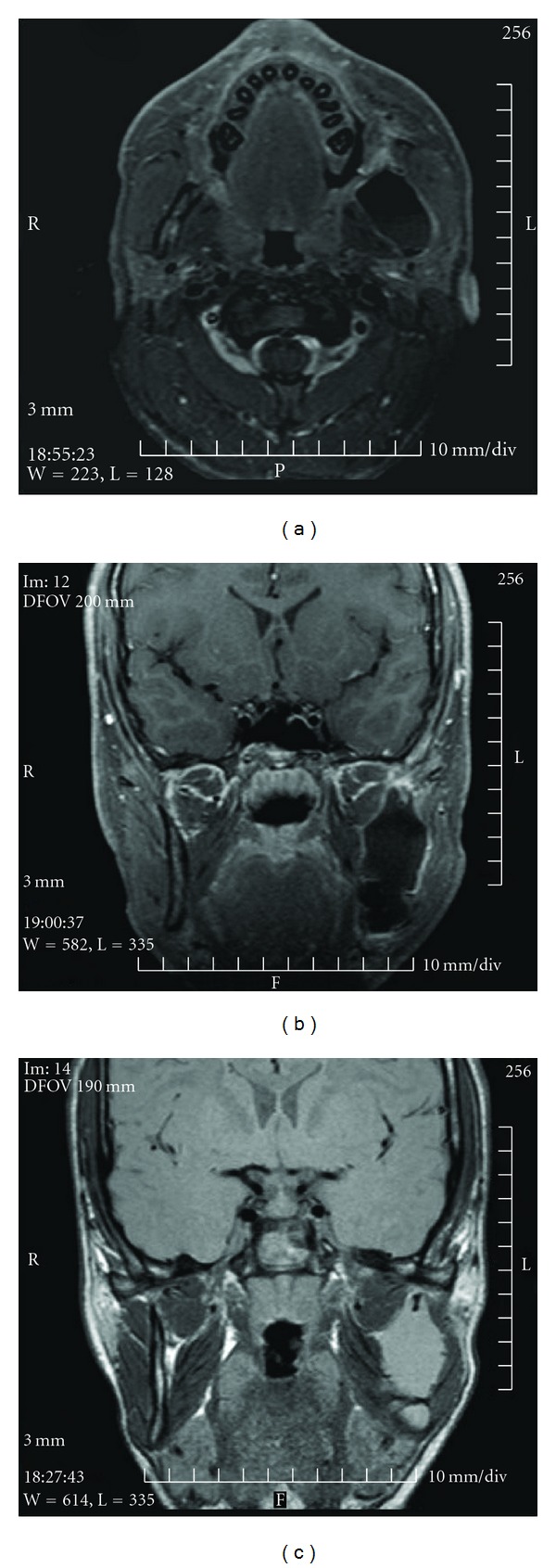
MR images demonstrate the low signal intensity on T1-weighted axial (a) and sagittal (b) images and the high signal intensity on T2-weighted sagittal images (c).

**Figure 7:**
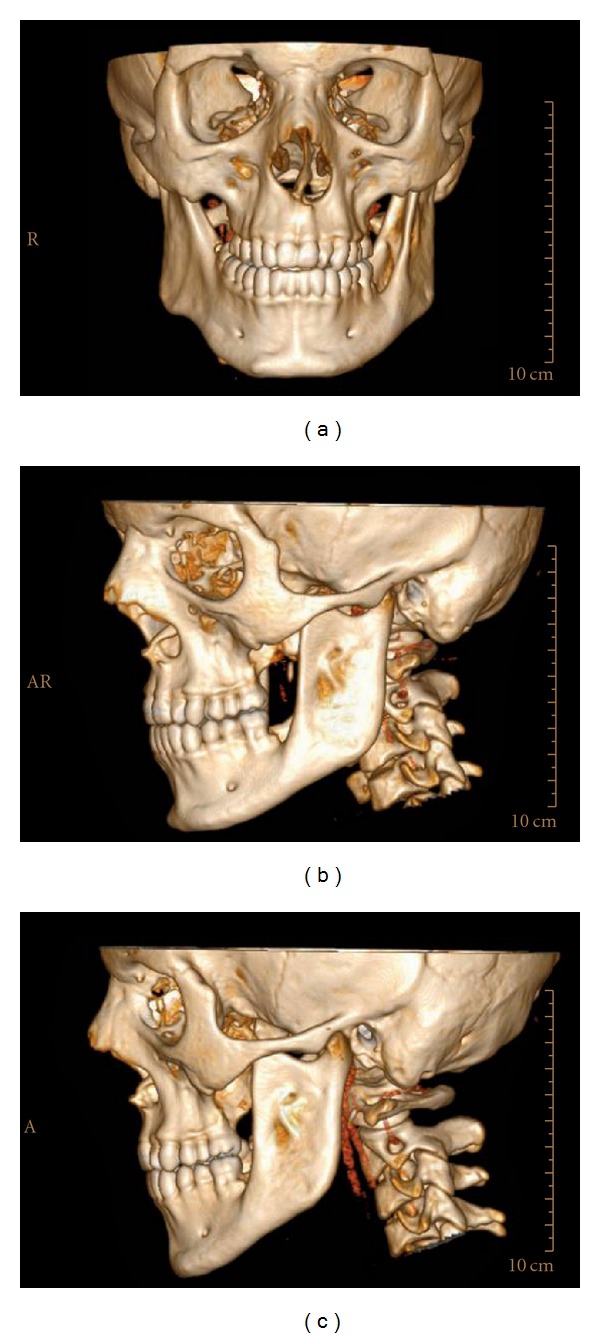
3D CT frontal (a) sagittal (b,c) images demonstrates the disappearance of a multilocular bony defect after 1 year follow up.

**Figure 8:**
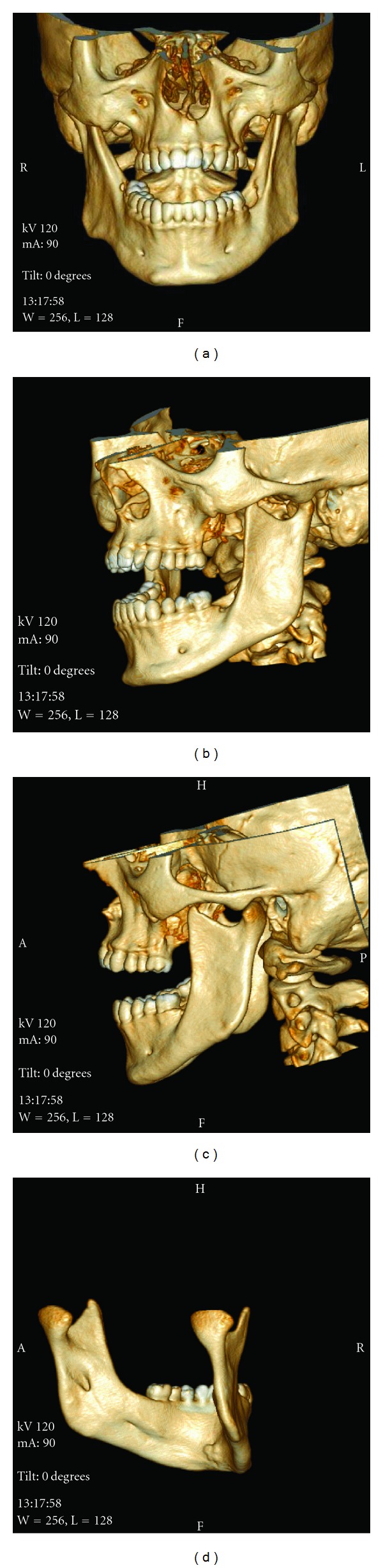
3D CT frontal (a) sagittal (b,c) and lingual (d) images demonstrate the normal bony structure of mandible in all direction after 3 year follow up.

The treatments of KCOTs were 18 (41.9%) for group 1 (Figure 1), and 10 (23.3%) group 2 (Figure 2), and 15 (34.8%) group 3 (Figure 3, 4, 5, 6, 7, and 8) (Table 6). The most treatment choices were used as enucleation in unilocular appearance and marsupialization in multilocular lesions were 17 (54.8%) and 8 (66.7%), respectively. A statistically significant relationship was found between the radiographic appearance and treatment methods (*P* = 0.00). No recurrence was found on 40.54 ± 23.02 months follows up.

## 4. Discussion

KCOT is one of the most commonly encountered odontogenic entities and requires special consideration because of its known aggressive behavior and tendency to recur. Clinical evidence of its known aggressive behavior is supported by reported cases penetrating the cortical bone and involving adjacent soft tissues, as well as extending to the skull base from the mandible or to the orbit and infratemporal fossa from the maxilla [25–28].

The sex distribution in this study was quite similar those of other previous studies, and we confirmed a male predominance of approximately 59%, as reported previously. The age distribution in our series was in agreement with those in other reports, with a peak incidence in the second decade of life, followed by the third decade; however, Kakarantza-Angelopoulou and Nicolatou found a major peak of frequency in the fifth and sixth decades of life in Greek patients [12].

The mandible is involved more frequently than the maxilla, and the percentage of KCOT occurring in the mandible ranges from 65% to 83% [11]. In our series the mandible was affected in 76.7% of lesions, and the most common site was the molar region (62.8%). The posterior regions of the mandible and maxilla were the most commonly affected parts of jaws, the findings being in close agreement with those of other reports [13, 29, 30].

The common radiographic features are unilocular or multilocular well-circumscribed radiolucent lesions surrounded by a thin sclerotic border. When unilocular radiolucent KCOT is encountered, it is difficult to distinguish it from other odontogenic or nonodontogenic cysts; when the multilocular variant is present, it is difficult to differentiate it from other odontogenic or nonodontogenic neoplasms (e.g., ameloblastoma, myxoma). KOCTs can be located at the periapical region of teeth, thus resembling periapical cysts; or they may envelope the crowns of unerupted teeth, mimicking dentigerous cysts; [3, 31] or they can be sited between the roots of the teeth, simulating lateral periodontal cysts or lateral radicular cysts; [32] or they can be located at the maxillary midline, suggestive of a nasopalatine duct cyst [33]. Radiographically, large KCOTs in the mandible can be indistinguishable from cystic ameloblastomas [34]. One radiographic feature that may suggest the diagnosis of KCOT is that KCOTs tend to grow in an anterior-posterior direction within the medullary cavity of the bone without causing obvious bone expansion. However, this feature is difficult to see in maxillary cysts. These lesions expand at the expanse of the medullary space. In maxillary lesions perforation of the floor of the maxillary sinus or the nasal cavity and the buccal cortex may occur [35]. Conventional radiographic imaging, such as panoramic and intraoral periapical films, is usually adequate to determine the location and estimate the size of a KCOT. With larger lesions, CT scans are required. CT scans of KCOTs show 3D extensin, sharply defined borders, and contents of water [36]. The MRI finding of KCOTs is described as uniformly thin walls with weak enhancement and fluids of heterogenous signal intensity. The contents of the cysts frequently showed intermediate or high T1-weighted signal intensity or intermediate T2-weighted signal intensity. In multilocular and large lesions of our series for detecting hard- and soft-tissue involvement of cysts, T-1 weighted MR image showed the lesion with thick, strongly enhanced walls of uniform thickness and heterogeneous fluid contents in T-2 weighted MR image. MR imaging features were differentiated from ameloblastomas which have homogeneous fluid contents [37].

Treatment of KCOT remains controversial. KCOT treated with enucleation had a significantly higher recurrent rate than those treated with other methods [26, 29, 30, 38]. Based on the high rate of recurrence, most authors advocate radical enucleation for small unilocular keratocysts and suggest resection and bone grafting for very large lesions. But there is a general agreement that complete removal of large multilocular KCOTs of the mandible ramus may be difficult because of the possibility that remnants of cystic tissue or that satellite microcysts may be left behind. The involvement of the condylar process of the mandible may require even a disarticulation and then reconstruction with bone grafts causing aesthetics and functional damages that, especially among young patients, could give the patient a poor quality of life [4, 38, 39].

 Most authors have shown the successful treatment of large KCOTs using the technique of decompression and irrigation. The benefits of this protocol over more conventional approaches (enucleation, en bloc resection) lie in the minimal surgical morbidity. In addition, the associated structures such as the inferior alveolar nerve and developing teeth are less vulnerable to damage. Forsell and Sainio showed a distinct change from parakeratinized group to orthokeratinized group and, in 4 cases, residual epithelium that could no longer be classified as KCOT [40]. Pogrel and Jordan recently reported 10 cases of nonsyndromic KCOTs treated with marsupialization that demonstrated complete resolution both clinically and radiographically after a mean follow up of 2.8 years [41]. Many questions remain as to the most appropriate treatment for the KCOTs. Morgan et al. reported that treatment with Carnoy's solution did not show a significant association with recurrence [8]. Voorsmith et al. reported a decreased recurrence rate following treatment with enucleation and Carnoy's solution (%2.5) compared with enucleation alone [42]. In our series, merely enucleation was performed in 18 (42%), enucleation followed by Carnoy's solution was performed in 10 (23.2%), marsupialization was performed in 15 (34.8%) of the KCOT cases as treatment. There was no statistical significance in recurrent rate following the different surgical modalities which corresponded with other reports.

 Inflammation in KCOT is associated with transition of the classic parakeratinized epithelium towards nonkeratinizing squamous epithelium [15, 29]. Inflammation has also been found to affect the proliferative potential of the epithelial lining of KCOT [43, 44]. Inflammation was detected histopathologically in 14 (32.5%). All inflammation was associated with parakeratocysts. The presence of one or more daughter cysts adjacent to the cystic wall of the tumour was demonstrated in 13% of lesions, which is considerably lower than the figure by Myoung et al. (30.1%). They advocate that KCOTs histopathologically that have satellite cysts have a high recurrence rate [4]. No reccurrence was observed in 5 KCOTs that have satellite cysts, and we suggest KCOT with satellite cysts must have longer follows up period.

Different views on the recommended duration of radiologic and clinical follow up are reported in the literature. Most reports point out that recurrence will appear within the 5 to 7 years [21, 30, 40]. In contrast, Crowley et al. found that %25 of their tumors recurred 9 or more years after initial treatment [13]. Vedtofte and Praiterious recommended 10 years of follow up [45]. Our follows up period was mean 28.49 ± 22.84 months and there were no recurrences in this period.

In conclusion, currently, the novel designation of the KCOT as a tumor and the research that influenced this change should serve as a compass by which clinicians can navigate future treatment plans. Recent advances in genetic and molecular research have led to increased knowledge of KCOT pathogenesis which hints at possible new treatment options [46]. As a result in our study we propose that successful treatment methods of these cysts are enucleation and Carnoy's solution in small lesions and marsupialization in lesions that have reached a very large size, but because KCOT is observed in second decande mostly, long-term follows up are suggested.

## Figures and Tables

**Table 1:** Patients characteristics.

Age	Sex		Localization		Radiographic features		Microscopic findings		
	♀	♂	Maxilla	Mandible	Multilocular	Unilocular	Para	Orto	Orto-para
40.59 ± 17.39	**16 **	**23 **	**10 **	**33 **	**12 **	**31 **	**38 **	**4 **	**1 **
	(41%)	(59%)	(23.3%)	(76.7%)	(28%)	(72%)	(88.4%)	(9.3%)	(2.3%)

**Table 2:** Clinical and radiologic diagnosis of lesion before histopathology.

Preclinical diagnosis of lesions		
Keratocystic odontogenic tumor	17	(39,5%)
Radicular cyst	14	(32,6%)
Dentigerous cyst	9	(21%)
Odontogenic myxoma	1	(2,3%)
Lateral periodontal cyst	1	(2,3%)
Globulomaxillary cyst	1	(2,3%)

Total	43	(100%)

**Table 3:** The distribution of the radiologic and microscopic findings of OKCs according to the age groups.

Age	Multilocular	Unilocular	Orthokeratocyst	Parakeratocyst	Ortho-parakeratocyst
	*N* = 12	*N* = 31	*N* = 4	*N* = 38	*N* = 1
10–19	—	2 (6.5%)	1 (25%)	1 (2.6%)	—
20–29	5 (41.6%)	9 (29%)	1 (25%)	13 (34%)	—
30–39	3 (25%)	5 (16%)	1 (25%)	7 (18.4%)	—
40–49	1 (8.3%)	6 (19.4%)		8 (21%)	—
50–59	2 (16.8%)	4 (12.9%)		4 (10.5%)	1 (100%)
60–69	1 (8.3%)	2 (6.5%)		3 (7.9%)	—
>70	—	3 (9.7%)	1 (25%)	2 (5.5%)	—

**Table 4:** The distribution of localization of KCOTs according to the radiographic features.

Sites	Multilocular	Unilocular
	*N* = 12	*N* = 31
Maxilla		
* Anterior premolar *(*N* = 8) (18.6%)	—	8 (25.8%)
* Posterior *(*N* = 2) (4.6%)	1 (8.3%)	1 (3.4%)

Mandible		
* Anterior premolar *(*N* = 6) (14%)	1 (8.3%)	5 (16%)
* Molar ramus *(*N* = 27) (62.8%)	10 (83%)	17 (54.8%)

**Table 5:** The distribution of microscopic findings of KCOTs according to the radiographic features.

Microscopic findings	Multilocular	Unilocular
	*N* = 12	*N* = 31
Orthokeratocyst	2 (16.7%)	2 (6.5%)
Parakeratocyst	9 (75%)	29 (93.5%)
Ortho-parakeratocyst	1 (8.3%)	**— **

**Table 6:** The distribution of treatment groups according to the localization of KCOTs in jaws.

Localization of KCOTs	Enucleation *N* = 18	Enucleation with Carnoy's solution *N* = 10	Marsupilizasyon *N* = 15	Total
*Maxilla*	—			
Anterior	7 (87.5%)	—	1 (22.5%)	** 8**
Posterior	1 (50%)	1 (50%)	—	** 2**
*Mandible*				
Anterior	2 (40%)	2 (40%)	1 (20%)	** 5**
Posterior molar and Ramus	8 (28.6%)	7 (25%)	13 (46.4%)	** 28**
* Unilocular *	17 (54.8%)	7 (22.6%)	7 (22.6%)	** 31**
* Multilocular *	1 (8.3%)	3 (25%)	8 (66.7%)	** 12**
